# MicroRNA-146a protects against myocardial ischaemia reperfusion injury by targeting Med1

**DOI:** 10.1186/s11658-019-0186-5

**Published:** 2019-11-27

**Authors:** Tiantian Zhang, Yiwen Ma, Lin Gao, Chengyu Mao, Huasu Zeng, Xiaofei Wang, Yapin Sun, Jianmin Gu, Yue Wang, Kan Chen, Zhihua Han, Yuqi Fan, Jun Gu, Junfeng Zhang, Changqian Wang

**Affiliations:** 1 Department of Cardiology, Shanghai Ninth People’s Hospital, Shanghai Jiao Tong University School of Medicine, Shanghai, 200011 China; 2 Department of Anaesthesiology, Shanghai Ninth People’s Hospital, Shanghai Jiao Tong University School of Medicine, Shanghai, 200011 China; 3 Department of Cardiovascular Surgery, Renji Hospital, School of Medicine, Shanghai Jiao Tong University, Shanghai, 200127 China

**Keywords:** Myocardial ischaemia reperfusion injury, Apoptosis, microRNA-146a, Med1

## Abstract

**Background:**

Myocardial ischaemia reperfusion injury (MIRI) is a difficult problem in clinical practice, and it may involve various microRNAs. This study investigated the role that endogenous microRNA-146a plays in myocardial ischaemia reperfusion and explored the possible target genes.

**Methods:**

MIRI models were established in microRNA-146a deficient (KO) and wild type (WT) mice. MicroRNA-146a expression was evaluated in the myocardium of WT mice after reperfusion. The heart function, area of myocardium infarction and in situ apoptosis were compared between the KO and WT mice. Microarray was used to explore possible target genes of microRNA-146a, while qRT-PCR and dual luciferase reporter assays were used for verification. Western blotting was performed to detect the expression levels of the target gene and related signalling molecules. A rescue study was used for further testing.

**Results:**

MicroRNA-146a was upregulated 1 h after reperfusion. MicroRNA-146a deficiency decreased heart function and increased myocardial infarction and apoptosis. Microarray detected 19 apoptosis genes upregulated in the KO mice compared with the WT mice. qRT-PCR and dual luciferase verified that Med1 was one target gene of microRNA-146a. TRAP220, encoded by Med1 in the KO mice, was upregulated, accompanied by an amplified ratio of Bax/Bcl2 and increased cleaved caspase-3. Inhibition of microRNA-146a in H9C2 cells caused increased TRAP220 expression and more apoptosis under the stimulus of hypoxia and re-oxygenation, while knockdown of the increased TRAP220 expression led to decreased cell apoptosis.

**Conclusions:**

MicroRNA-146a exerts a protective effect against MIRI, which might be partially mediated by the target gene Med1 and related to the apoptosis signalling pathway.

## Background

Myocardial ischaemia reperfusion injury (MIRI) is a double-edged sword for myocardial infarction patients [1, 2]. With the opening of blocked blood vessels, endangered myocardium can be saved; however, the reperfusion causes numerous free radicals and calcium overload, leading to a certain degree of myocardial injury. MIRI, which is the damage caused by reperfusion [1, 2], is undoubtedly an obstacle for the application of reperfusion in clinical practice. Data have shown that 5–6% of patients have cardiovascular events within 30 days after myocardial ischaemia reperfusion (MIR), which not only causes great harm to patients but also creates a heavy social burden [1]. Thus, in-depth study of the occurrence, development and effective intervention measures of MIRI has become an urgent need to solve the problem.

The mechanisms of MIRI are complex and may involve various microRNAs playing protective roles, harmful roles, or double-sided roles in different stages [3–8]. For example, in vivo study has shown that inhibition of increased microRNA-24 in the infarction area can alleviate MIRI by preventing the apoptosis of cardiomyocytes [5, 6], whereas injection of exogenous MicroRNA-24 analogues inhibited cardiomyocyte apoptosis, thereby reducing myocardial infarct size and cardiac dysfunction [5, 7, 8]. MicroRNA-21 and microRNA-29 also play dual roles in MIRI [4, 5, 9, 10].

In our previous study, we detected the expression of microRNAs in the plasma of myocardial infarction patients who experienced percutaneous coronary intervention, and we found that microRNA-146a was one of the microRNAs that increased after reperfusion. Thus, we were interested in the role that microRNA-146a plays in MIRI. One study has reported that at 7 days before ischaemia, the injection of exogenous microRNA-146a analogues into mice myocardium was able to decrease inflammation in MIRI by targeting the TLR4-IRAK1-TRAF6 pathway [11]. However, no data have shown the expression of microRNA-146a in myocardium after MIR within 0–24 h and what will happen if endogenic microRNA-146a is lost. Furthermore, whether microRNA-146a can influence MIRI by targeting other genes or other pathways is still unclear. Therefore, this study aimed to clarify the expression and role of endogenic microRNA-146a and other mechanisms of microRNA-146a in MIRI, which will provide new insight into the treatment of MIRI.

## Material and methods

### Animal care

Global microRNA-146a deficient (microRNA-146a−/−, KO) mice were obtained from Jackson Laboratory, while wild-type (WT) C57BL/6 mice genetic background control mice were obtained from Shanghai SLAC Experimental Animal Co., Ltd. They were maintained in the division of Laboratory Animal Resources under specific pathogen free conditions. After the mice were bred, homozygous genotypes were identified and screened. Mice were euthanized with isoflurane over-anaesthesia. All animal experiments were approved by the Shanghai Ninth People’s Hospital institutional ethics committee (HKDL2017300) and were performed in accordance with the ethical standards outlined in the 1964 Declaration of Helsinki and its later amendments.

### In vivo model of MIRI in mice

Two-month-old male mice were randomly selected to be anaesthetized by 5% isoflurane inhalation, intubated and then connected to a rodent ventilator (model 683, Harvard Apparatus, Inc., USA) with 65% oxygen and anaesthesia with 3–5% isoflurane to maintain smooth breathing in the mice, without resistance and pain responses. After the chest was opened, MIR was induced by ligating the left anterior descending (LAD) artery on the exposed heart with 7–0 silk ligature for 30 min, followed by pulling on the exteriorized suture to release the knot for reperfusion. Regional ischaemia was confirmed by ECG changes (ST elevation). After reperfusion for appropriate time, hearts were harvested for qRT-PCR, Evans Blue/TTC staining, TUNEL, Gene Chip and Western blotting. Echocardiography was performed on the first and third day after reperfusion. WT and KO mice that had not experienced ligation and loosening were reviewed as sham controls.

### Quantitative real-time PCR (qRT-PCR) of microRNA-146a and target genes

Total RNA (including microRNA) extraction of H9C2 cells transfected with microRNA-146a inhibitor (miR20000852, RN:R10034.5, Ribobio, China) and myocardium at risk, including ischaemia and infarction tissues, using TRIZOL reagent (Qiagen) was performed in accordance with the manufacturer’s protocol, followed by reverse transcription with the Thermo Cycler machine (Applied Biosystems). qRT-PCR was conducted using a real-time PCR machine (LightCycler 480 II, Roche). The microRNA-146a level was quantified by qRT-PCR using a specific assays for microRNA (Applied Biosystems, USA) and TaqMan Universal Master Mix (Applied Biosystems, USA). Specific primers for microRNA-146a were obtained from Applied Biosystems [primer identification numbers: 000468 for microRNA-146a and 001973 for U6 small nuclear RNA (snRU6)]. mRNA of the possible target genes was quantified using Takara reverse transcription assay (R0037A) and SYGRII (RR820A). Specific primers were synthesized from Sangon Biotech (Shanghai, China). The mRNA levels were quantified with the 2^-ΔΔcp^ method.

### Echocardiography

Echocardiography was performed using a Vevo 770 high-resolution imaging system on the first and third day after myocardial ischaemia reperfusion. Two-dimensional and M-mode echocardiogram images were obtained after the animals were anaesthetized with isoflurane. Ejection fraction (EF) and fractional shortening (FS) were measured to evaluate the heart function.

### Assessment of area at risk and infarct size

After reperfusion for 4 h, the chest was opened again to expose the heart. The LAD was re-ligated at the previous site of ligation for staining with 1% Evans Blue from the abdominal aorta until the whole heart turned blue. Then, the left ventricle was harvested and washed with saline. Each left ventricle was then sliced horizontally (i.e., five slices). All slices were incubated in 1% TTC for 15 min at 37 °C and then fixed in 4% neutral buffered formalin for 24 h. The white infarction and red risk areas were determined using an image analyser, corrected for the weight of each slice to sum for one whole heart. The ratios of the area at risk (AAR) to the left ventricle area (LV) and infarction size (IS) to AAR were calculated and expressed as a percentage to perform statistical analysis.

### Terminal deoxynucleotidyl transferase dUTP nick end labelling (TUNEL) analysis of in situ apoptosis

At the end of reperfusion for 2 h, the hearts were fixed with 4% buffered paraformaldehyde through abdominal aorta injection. Then, the hearts were sliced horizontally, and embedded in paraffin to yield several 5 μm thickness sections. These sections were incubated with a prepared labelling mixture supplied by the assay (Roche, USA) at 37 °C for 1 h. The nuclei of living and apoptotic cells were counterstained with Hoechst 33342 (Invitrogen). Then, 40× and 400× magnification images were taken using a microscope (Nikon). The number of apoptotic cardiomyocytes was examined with the image processing software IPP6 and presented as the percentage of total cells counted.

### Microarray

The Agilent Sure Print G3 Mouse GE V2.0 Microarray (8*60 K, Design ID,074809) was used. Total RNA was quantified, and the RNA integrity was assessed. The sample labelling, microarray hybridization and washing were performed based on the manufacturer’s standard protocols. After washing, the arrays were scanned using the Agilent Scanner G2505C (Agilent Technologies). Feature Extraction software (version 10.7.1.1, Agilent Technologies) was used to analyse array images to obtain raw data. Genespring (version 13.1, Agilent Technologies) was used to complete the analysis with the raw data. The threshold set for the up- and down-regulated genes was a fold change ≥2.0 and a *P* value≤0.05. The microarray experiments were performed at Shanghai OE Biotech. Co., Ltd. (Shanghai, China).

### Dual luciferase reporter assay

Two hundred and ninety three T cells were cultured in 24-well plates and transfected with PGL3 luciferase reporter plasmids containing wild-type or mutated mediator complex subunit 1 (Med1) 3′UTR and microRNA-146a (Genechem) using Lipo3000 reagent (Invitrogen). Cells were harvested 24 h later for luciferase activity detection using the Dual-Luciferase Reporter Assay System (Promega), according to the manufacturer’s protocol.

### Western blotting

After 2 h of reperfusion, hearts were harvested. Total protein extracted from ischaemic heart tissues with RIPA was separated by SDS-polyacrylamide gel electrophoresis and transferred onto PVDF membranes (Millipore). After being blocked with milk, the membranes were incubated with the primary antibodies anti-TRAP220 (Bethyl), anti-Bcl2 (CST), anti-Bax (CST), and anti-cleaved caspase-3 (CST) overnight, followed by incubation with peroxidase conjugated secondary antibodies. Analysis was conducted using the ECL system (Fusion FX7).

### Construction of Lenti-Med1 RNAi

A linearized vector was obtained through digestion with restriction enzymes. Primers were annealed to prepare the desired fragment, and enzyme sites were added to the ends. Then, the vector was connected to the desired fragment that contained the same restriction sites with one another at the ends. Competent cells were transfected with the product obtained, and the monoclonal ones were selected for identification, sequencing and analysis. The correct one was expanded and extracted to obtain high-purity plasmids for virus packaging. 293 T cells were transfected with plasmids to obtain the target virus. After the enrichment, purification and quality inspection of virus, the construction of Lenti-Med1-RNAi was completed.

### Rescue study

H9C2 cells were cultured in 6-well plates and transfected with microRNA-146a inhibitor using lipo3000 for 48 h to inhibit the expression of microRNA-146a and increase the expression of TRAP220, which was encoded by the Med1 gene. In addition, the cells were infected with Lenti-Med1 RNAi for 48 h to decrease the expression of TRAP220. qRT-PCR and Western blotting were applied to verify the effect. With the treatment above, H9C2 experienced hypoxia and re-oxygenation in a hypoxia culture chamber. After that, the apoptosis of H9C2 was detected with flow cytometry using Annexin V, FITC Apoptosis Detection Kit (Dojindo) according to the manufacturer’s protocol.

### Statistical analysis

Quantitative data were presented as the mean ± standard deviation. Statistical significance was determined via the independent sample t test between groups or ANOVA in multiple groups with SPSS 21.0 software. *P* < 0.050 was considered statistically significant.

## Results

### MicroRNA-146a was up-regulated at an early stage of MIR

**Fig. 1:**
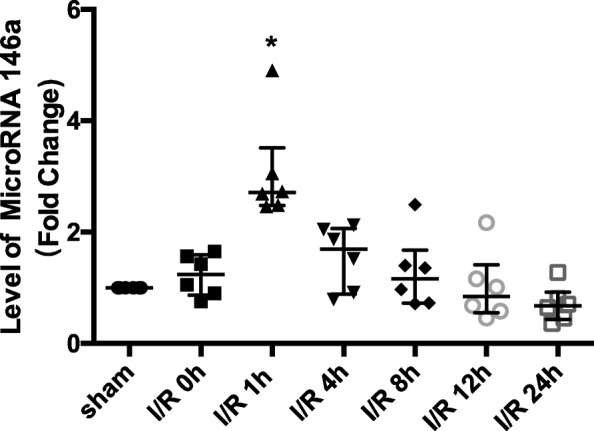
MicroRNA-146a was upregulated at an early stage of myocardial ischaemia reperfusion injury (MIRI) in vivo. MicroRNA-146a expression was detected through qRT-PCR at different times after myocardial ischaemia reperfusion (MIR). Compared with the sham control group, *P < 0.05, *n* = 6

To demonstrate the expression of microRNA-146a in MIRI, WT mice were applied to build MIRI models in vivo. At different times of reperfusion, the expression of microRNA-146a was detected with qRT-PCR. We found that in the first hour after reperfusion, the expression of microRNA-146a was increased, but it declined slowly to the previous level in the next 23 h (*P* = 0.046), Fig. 1.

### MicroRNA-146a deficiency increased MIRI

#### MicroRNA-146a deficiency reduced cardiac function in MIRI

**Fig. 2:**
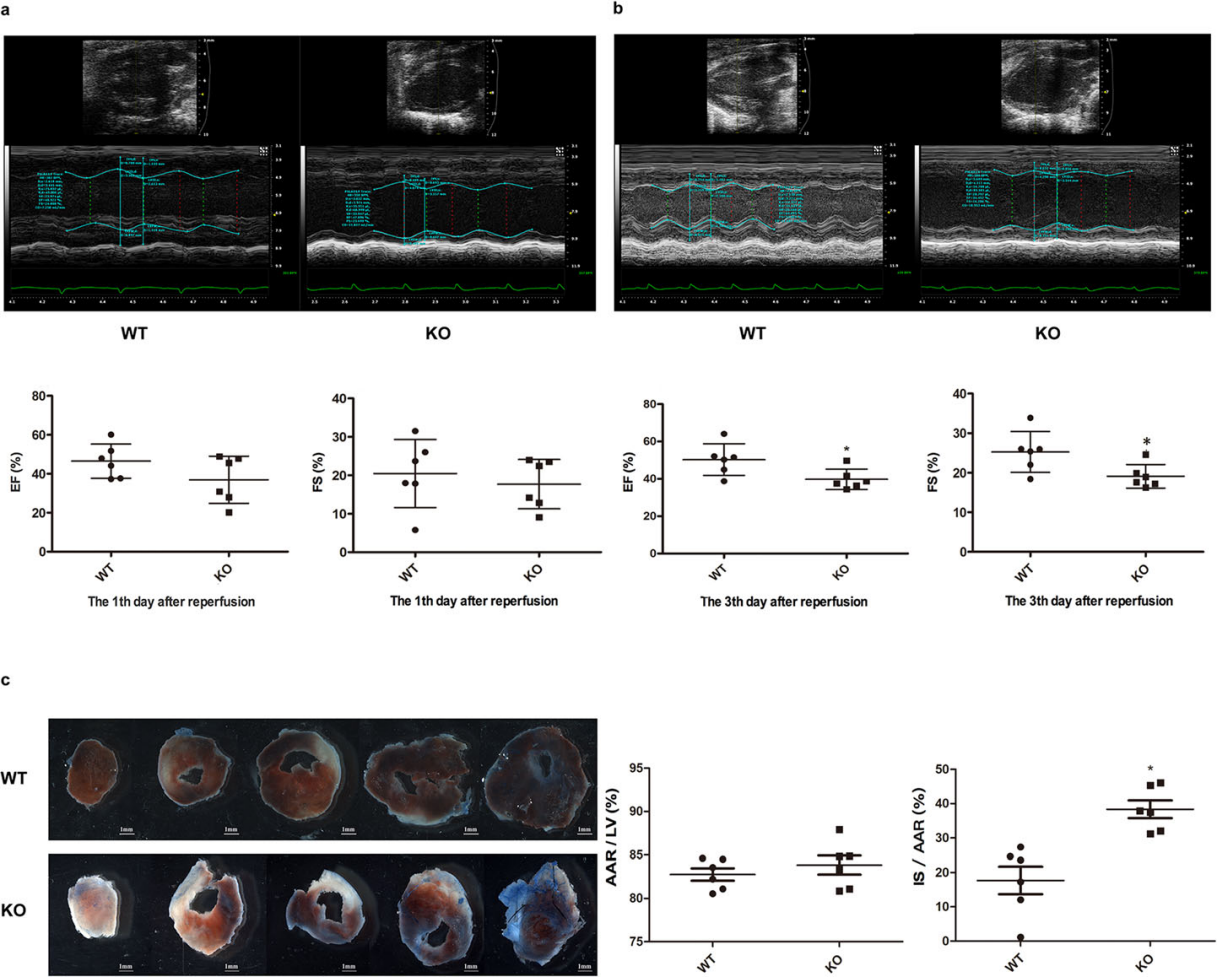
MicroRNA-146a deficiency increased MIRI, **a**, **b**. MicroRNA-146a deficiency reduced cardiac function. Echocardiography was used to examine the heart function of WT and KO mice after MIR at the first and third days, EF and FS were detected. Compared with WT, *P < 0.05, *n* = 6. **c** MicroRNA-146a deficiency increased the myocardial infarct size. WT and KO mice were subjected to I/R, and then AAR and infarct size were subsequently measured using Evans Blue/TTC staining (10× magnification). WT, wild type; KO, microRNA-146a−/−; IR, ischaemia reperfusion; EF, ejection fraction; FS, fractional shortening; TTC, triphenyltetrazolium chloride; AAR, area at risk; IS, infarction size. Compared with WT, *P < 0.05, *n* = 6

To explicate the function of endogenous microRNA-146a in MIRI, we built the MIRI model in vivo with KO mice and WT mice, and examined cardiac function using echocardiography at the first and third day after reperfusion. As shown in Fig. 2a, no distinction of EF (*P* = 0.149) and FS (*P* = 0.546) on the first day was found. However, the deficiency of microRNA-146a led to the decline of EF (*P* = 0.028) and FS (*P* = 0.030) on the third day after reperfusion (*P* < 0.05), Fig. 2b.

#### MicroRNA-146a deficiency increased myocardial infarct size in MIRI

We also checked the infarct and risk size of myocardium after ischaemia reperfusion in KO mice compared with WT mice. Figure 2c showed that the AAR/LV in KO mice and WT mice were similar (*P* = 0.426), which demonstrated that the models were successful, and the two groups were comparable. However, IS/AAR in KO mice was much higher than that in WT mice (*P* = 0.002), meaning that microRNA-146a deficiency led to more injury in myocardial ischaemia reperfusion.

### MicroRNA-146a deficiency increased apoptosis in MIRI

**Fig. 3:**
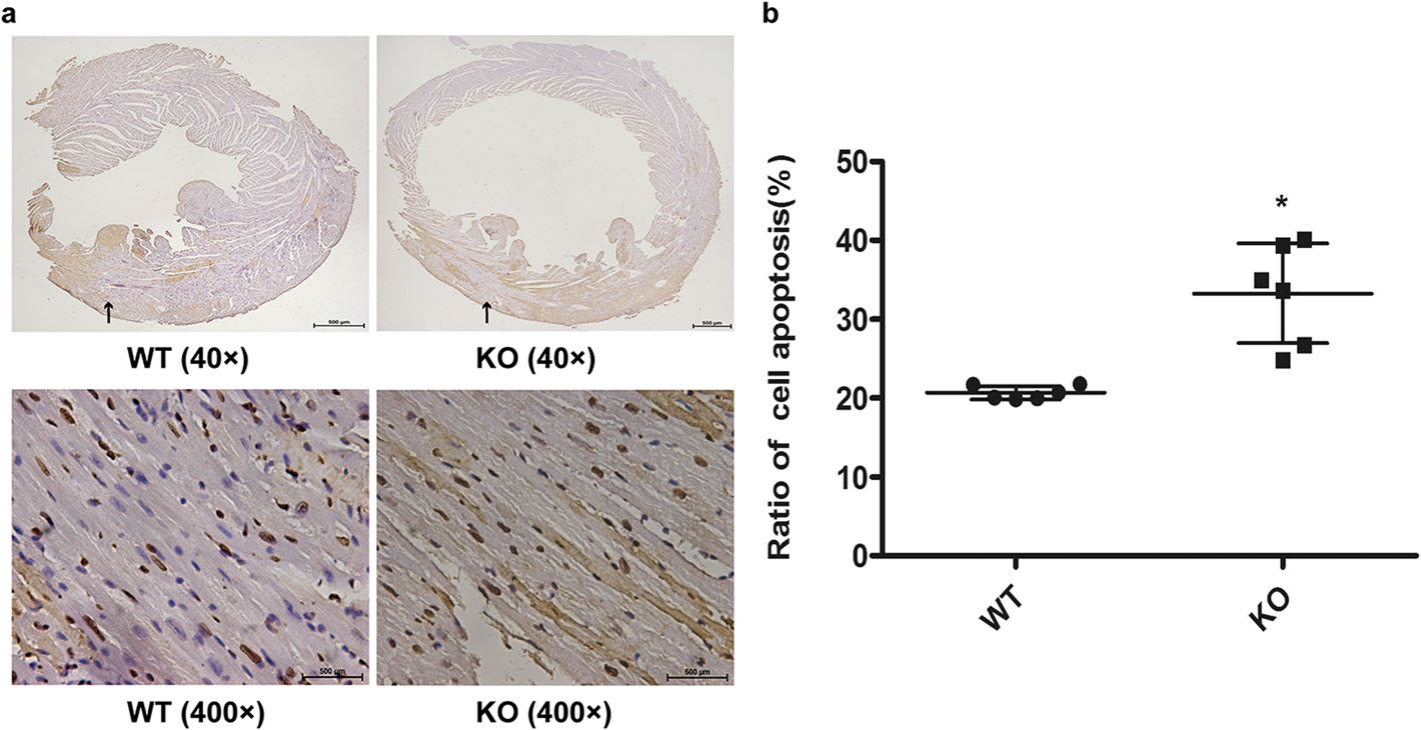
MicroRNA-146a deficiency upregulated cardiomyocyte apoptosis in vivo after MIR. **a** WT and KO mice were subjected to I/R, and in situ apoptosis was determined using a TUNEL assay (Panorama above: 40× magnification; zoom below: 400× magnification). **b** An independent sample t test was performed for the comparison of the two groups. Compared with WT, *P < 0.05, *n* = 6

Apoptosis was the main cause of myocardial injury, so we measured in situ apoptosis of cardiomyocytes in vivo with TUNEL. Figure 3a and Fig. 3b showed that the number of apoptosis cells in the KO group was far higher than that in the WT group after MIR (*P* = 0.001).

### Possible target genes of microRNA-146a in MIRI

#### 19 apoptosis related genes were detected to be the possible target of microRNA-146a

**Fig. 4:**
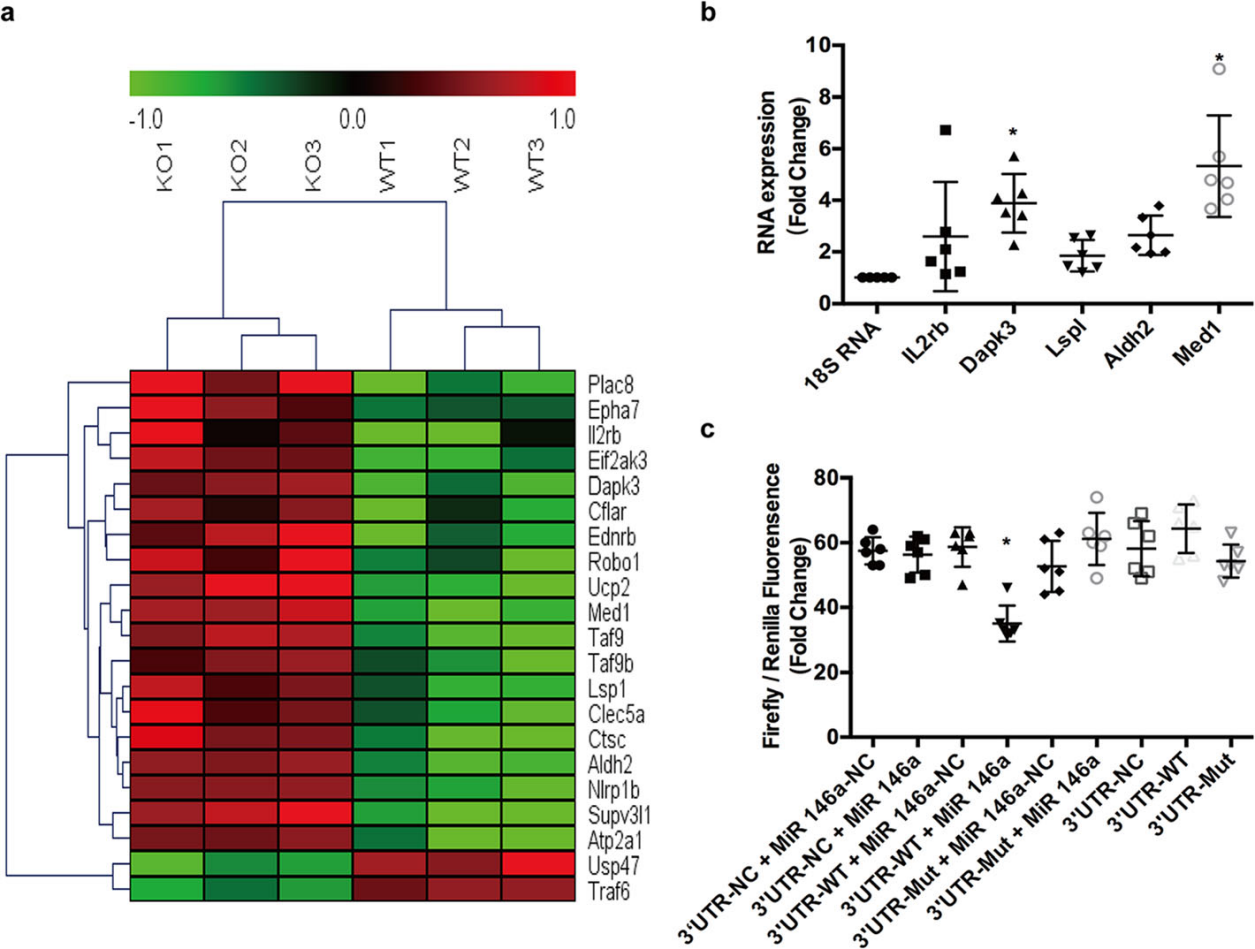
Med1 was one of the target genes of microRNA-146a. **a** WT and KO mice were subjected to MIR, and then possible target genes were checked with microarray. The heatmap showed 19 apoptosis related genes were up-regulated. **b** WT and KO mice were stimulated with MIR, and the mRNA levels of IL2rb, Dapk3, Lspl, Aldh2, and Med1 were detected through qRT-PCR, with 18 s RNA as an internal reference. Compared with 18 s RNA, *P < 0.05, *n* = 6. **c** 293 T cells were transfected with PGL3 luciferase reporter plasmids containing wild-type or mutated Med1 3′UTR and microRNA-146a, using NC as controls. NC, negative control. Compared with other groups, *P < 0.05, *n* = 6

To explore new target genes of microRNA-146a, microarray was applied to distinct the difference of gene expression in myocardium between KO and WT mice after MIR. There were approximately 136 apoptosis related genes increased, including 19 genes predicted by TargetScan to be able to combine with microRNA-146a, which are shown in the heatmap in Fig. 4a.

#### MicroRNA-146a deficiency increased 5 apoptosis related genes’ mRNA expression after MIRI

With the preliminary results of microarray and TargetScan, we made further verification through qRT-PCR in myocardium of KO and WT mice that experienced myocardial ischaemia reperfusion. Figure 4b shows that, in the 19 apoptosis related genes, mRNA expression of 5 genes was upregulated: IL2rb (*P* = 0.323), Lsp1 (*P* = 0.875), Aldh2 (*P* = 0.295), Dapk3 (*P* = 0.009) and Med1 (*P* = 0.000). Med1 was the gene with the highest expression. Thus, it was the best candidate target gene for microRNA-146a in MIRI.

#### Med1 was one target gene of microRNA-146a

We chose Med1 with the highest mRNA expression to check if it was the target gene of microRNA-146a using dual luciferase reporter in 293 T cells. After transfection with PGL3 luciferase reporter plasmids containing wild-type or mutated Med1 3′UTR and microRNA-146a, as shown in Fig. 4c, the fluorescence intensity in the microRNA-146a + Med1 WT-3′UTR group was significantly lower than that in other groups (*P =* 0.000), demonstrating that Med1 was the target gene of microRNA-146a.

### MicroRNA-146a deficiency increased TRAP220, accompanied by aggravated apoptosis protein expression in myocardium

**Fig. 5:**
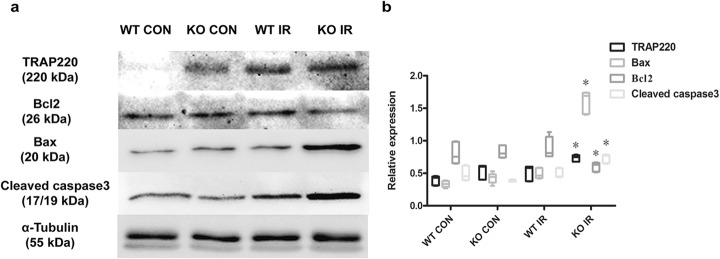
MicroRNA-146a deficiency increased TRAP220 accompanied with amplified ratio of Bax/Bcl2 and increased cleaved caspase-3 after IR. **a** WT and KO mice were subjected to I/R, and the protein expression levels of TRAP220, Bcl2, Bax, cleaved caspase-3 in myocardium were detected through Western blotting, with sham groups as the controls. **b** ANOVA was performed for the between-group comparisons. Compared with WT IR group, *P < 0.05, *n* = 6

To explore the possible mechanism of microRNA-146a and Med1 in MIRI, apoptosis related protein was detected through Western blotting analysis. As shown in Fig. 5a and b, we found that TRAP220 was increased when myocardium experienced MIR, compared with the sham control group (*P =* 0.002). Under the same condition of ischaemia reperfusion, myocardial microRNA-146a deficiency led to a greater increase of TRAP220, which was encoded by Med1, compared to WT. At the same time, apoptosis related protein Bax and cleaved caspase-3 were also upregulated (*P =* 0.000). However, the expression of Bcl2 was decreased (*P =* 0.004), which suggested that the lack of microRNA-146a could cause increased expression of TRAP220, accompanied by an amplified ratio of Bax/Bcl2 and increased cleaved caspase-3.

### MicroRNA-146a decreased the apoptosis of H9C2 with stimulation of hypoxia and re-oxygenation by targeting Med1

**Fig. 6:**
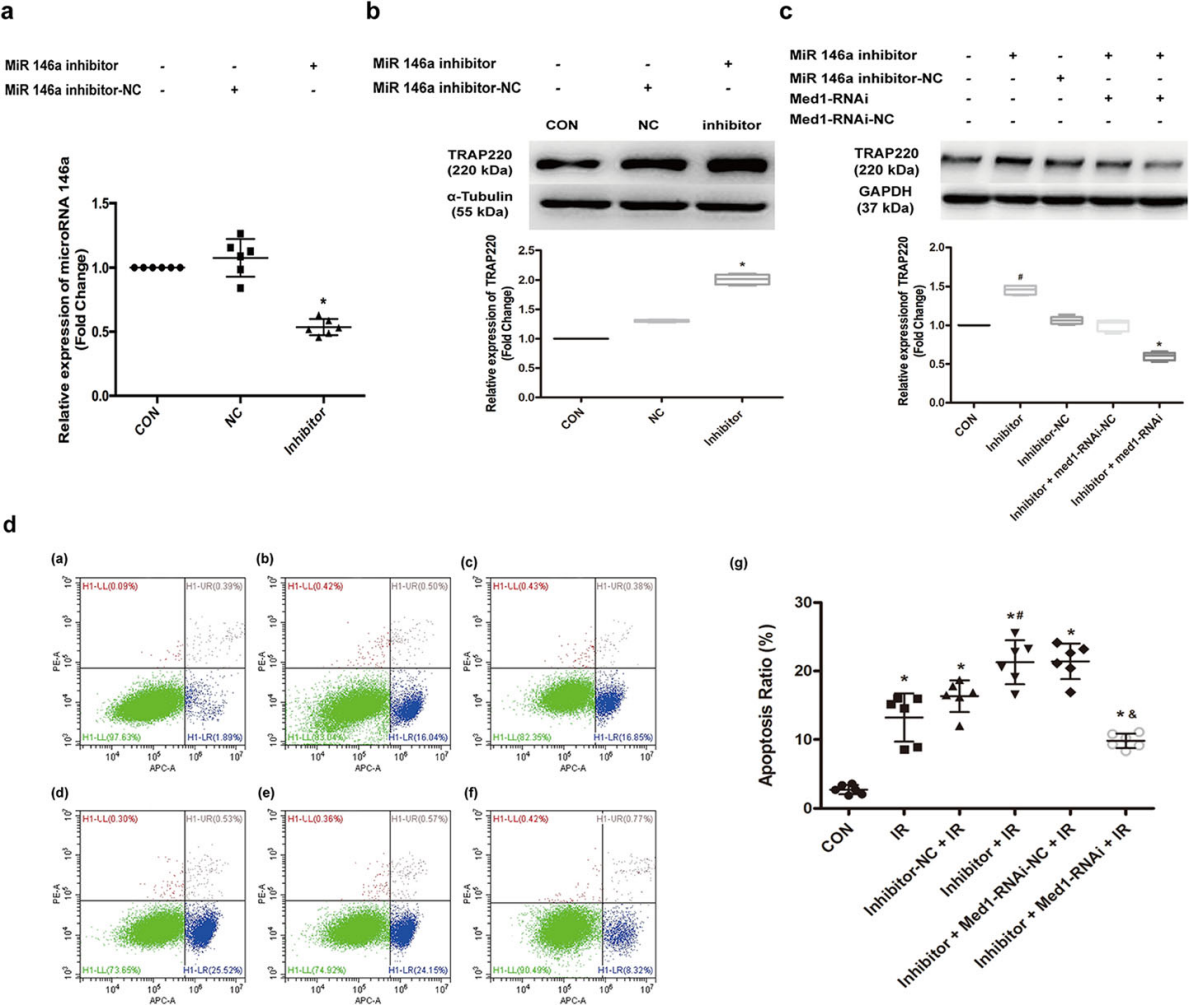
MicroRNA-146a decreased the apoptosis of H9C2 in stimulation of hypoxia and re-oxygenation by targeting Med1. **a** MicroRNA-146a inhibitor suppressed the expression of microRNA 146a in H9C2 cells. Compared with negative control group, *P < 0.05, *n* = 6. **b** MicroRNA-146a inhibitor increased the expression of TRAP220 encoded by Med1 gene, *P < 0.05, *n* = 6. **c** Med1-RNAi downregulated the expression of TRAP220 in H9C2 cells which was upregulated by microRNA-146a inhibitor. Compared with control group, ^#^*P* < 0.05, *n* = 6; Compared with Inhibitor group, *P < 0.05, *n* = 6. **d** MicroRNA-146a inhibitor increased the apoptosis of H9C2 after hypoxia and re-oxygenation, which was decreased by Med1-RNAi. d-(a). CON group: H9C2 cells; d-(b). IR group: H9C2 cells experienced hypoxia and re-oxygenation; d-(c). Inhibitor-NC + IR group: H9C2 cells were stimulated with microRNA-146a inhibitor negative control and then experienced hypoxia and re-oxygenation; d-(d). Inhibitor + IR group: H9C2 cells were stimulated with microRNA-146a inhibitor and then experienced hypoxia and re-oxygenation; d-(e). Inhibitor + Med1-RNAi-NC + IR group: H9C2 cells were treated with microRNA-146a inhibitor and then transfected with Med1-RNAi-negative control. Then, these H9C2 cells were stimulated with hypoxia and re-oxygenation; d-(f). Inhibitor + Med1-RNAi + IR group: H9C2 cells were treated with microRNA-146a inhibitor and then transfected with Med1-RNAi. Then, these H9C2 cells were stimulated with hypoxia and re-oxygenation. d-(g). ANOVA was performed for between-group comparisons in the experiment. Compared with CON group, *P < 0.05, *n* = 6; compared with Inhibitor-NC + IR group, ^#^*P* < 0.05, *n* = 6; compared with Inhibitor + Med1-RNAi-NC + IR group, ^&^*P* < 0.05, *n* = 6. CON: control group; NC: negative control; Inhibitor: microRNA-146a inhibitor; Med1-RNAi: Lenti-Med1 shRNA; Med1-RNAi-NC: Lenti-Med1 shRNA negative control

To further confirm whether microRNA-146a can influence apoptosis in MIRI partly through targeting Med1, a rescue study of H9C2 cells was conducted. As shown in Fig. 6a and b, we found that microRNA-146a inhibitor decreased the expression of microRNA-146a (*P =* 0.000) while it increased TRAP220 protein (*P =* 0.000) in H9C2. Lenti-Med1-RNAi successfully suppressed the upregulation of TRAP220 (*P =* 0.000) (Fig. 6c). The flow cytometry results (Fig. 6d) demonstrated that the apoptosis of H9C2, which experienced hypoxia and re-oxygenation, was greater than that of the control group (*P =* 0.000). When the expression of microRNA-146a was suppressed, the apoptosis ratio was further increased (*P =* 0.018). However, with the downregulation of Med1, after the inhibition of microRNA-146a in H9C2 cells that were stimulated with hypoxia and re-oxygenation, the apoptosis ratio decreased greatly (*P =* 0.000). This result suggested that microRNA-146a did decrease the apoptosis of H9C2 stimulated with hypoxia and re-oxygenation, partly by targeting Med1.

## Discussion

In the present study, we examined the expression and role of endogenous microRNA-146a in MIRI, and we explored the possible target genes and pathways. Our results indicated that microRNA-146a was upregulated at an early stage of MIR, and endogenic microRNA-146a deficiency increased MIRI, demonstrating a protective role in this process. The role of endogenic microRNA-146a was consistent with that of exogenous microRNA-146a mimics previously reported, which also proved that the increase of microRNA-146a in the first hour after reperfusion was a compensatory protection initiated by the body itself. However, this compensatory protective effect slowly disappeared after that. Therefore, upregulation of microRNA-146a occurs as early as the reperfusion occurs and as long as the injury duration would be beneficial for ischaemic myocardium. Thus, interventions that can increase microRNA-146a, such as drugs, surgery or direct exogenous addition of microRNA-146a mimics at a suitable time, can be used to protect ischaemic myocardium after reperfusion.

MicroRNA is a class of non-coding single-stranded RNA molecules encoded by endogenous genes of approximately 22 nucleotides [3, 12, 13]. They are capable of completely or incompletely binding to the target genes and then degrading them or modulating their functions [3, 12, 13]. It has been demonstrated that microRNA-146a can target many genes, such as IRAK1, IRAK2, TRAF6, RIG-I, IRF-5, STAT-1, PTC1, Numb, and WASF2, to play a variety of roles in human diseases, including cancers and inflammatory immune diseases [14–18]. IRAK1 and TRAF6 are the downstream molecules of the TLR-induced NF-κB signalling pathway, and they have often been identified as the target genes of microRNA-146a in inflammatory immune diseases containing MIRI [11, 19–21]. However, a few other target genes of microRNA-146a in MIRI are known. In this study, we found that mediator complex subunit 1 (Med1) was one target gene of microRNA-146a, and TRAP220 protein encoded by Med1 was upregulated when microRNA-146a was deficient.

The multi-subunit mediator is an evolutionarily conserved transcription co-regulatory nuclear complex in eukaryotes. It is needed for the transcription regulation of gene expression in general, as well as in a gene specific manner. Mediator complex subunits interact with different transcription factors and as components of the RNA Pol II transcription initiation complex; in doing so, they act as a bridge between gene specific transcription factors and general Pol II transcription machinery [22]. Med1 is also known as TRAP220 in mice and RB18A in humans. Researchers have reported that cardiac Med1 was necessary for survival in mice because it regulated cardiac metabolic and mitochondrial genes. Genetic deletion of Med1 resulted in embryonic lethality, largely due to impaired cardiac development [23, 24]. Other studies have also shown that cardiomyocyte-specific ablation of the Med1 subunit of the mediator complex might cause lethal dilated cardiomyopathy [25]. However, no data detailing whether Med1 can influence MIRI are available. In our study, we found that the upregulation of TRAP220 in microRNA-146a deficient mice that experienced myocardial ischaemia reperfusion was accompanied by an amplified ratio of Bax/Bcl2 and increased cleaved caspase-3. The rescue study verified that Med1 was indeed one target gene of microRNA-146a in MIRI.

Apoptosis is a sophisticated process that is the main cause of MIRI [26]. There are two main pathways involved in apoptosis: the extrinsic (death receptor) pathway and the intrinsic (mitochondrial) pathway [27, 28]. However, the last stage of both pathways is initiated by the cleavage of caspase-3 and results in DNA fragmentation, degradation of cytoskeletal and nuclear proteins, cross-linking of protein, formation of apoptotic bodies, expression of ligands for phagocytic cell receptors and finally, uptake by phagocytic cells. In the intrinsic pathway, stimuli, including radiation, toxins, viral infections, hyperthermia, hypoxia and free radicals, are able to cause changes in the inner mitochondrial membrane, which may result in an opening of the mitochondrial permeability transition pore and loss of the mitochondrial transmembrane potential. Then, two main groups of normally sequestered pro-apoptotic proteins are released from the intermembrane space into the cytosol, consisting of cytochrome c, Smac/DIABLO, and the serine protease HtrA2/Omi, which will activate the caspase-dependent mitochondrial pathway. Cytochrome c initiates activation of a series of kinases, including caspase-3, which is the ultimate executor of apoptosis [27, 28]. These apoptotic mitochondrial events can be regulated by the Bcl-2 family proteins, such as anti-apoptotic proteins Bcl-2 and pro-apoptotic proteins Bax, which control the release of cytochrome c from the mitochondria via alteration of mitochondrial membrane permeability [27, 28]. p53, a tumour suppressor protein, has a critical role in regulation of the Bcl-2 family [27–31]. The p53 tumour suppressor gene is a transcription factor that regulates the cell cycle. It can activate DNA repair proteins when DNA has sustained damage, hold the cell cycle at the G1/S regulation point on DNA damage recognition, and initiate apoptosis if the DNA damage proves to be irreparable. p53 is one regulator of Bcl2 and Bax [27–31]. Researchers have revealed that RB18A, a member of the Med1 family, self-oligomerized and interacted with the p53 protein in vitro when K562 (an erythroleukaemia cell line) and H1299 (pulmonary embryo carcinoma) cells, two human p53-null cell lines, were used to transfect with p53wt cDNA in the presence or absence of RB18A cDNA [29, 30]. In addition, with immunoprecipitation, they demonstrated that in vivo, RB18A directly interacted (through its C-terminal domain) with wild type p53 (p53 wt), acting as a cofactor of transcription by directly regulating p53-transactivating activity on the promoters of Bax, p21Waf1 and IGF-BP3 genes [29, 30], while RB18A alone did not affect cell apoptosis. In this process, RB18A activated Bax promoter and inhibited p21Waf1 and IGF-BP3 promoters [29, 30, 32]. TRAP220, a 220 KDa thyroid hormone receptor-associated protein, which shared 99% sequence identity within the RB18A coding sequence (with only minor sequence variations), was also demonstrated to interact with p53 wt as RB18A [29, 30, 33]. In our study, we inferred that the increased TRAP220 protein in the KO mice that experienced myocardial ischaemia reperfusion might interact with p53 protein, activate the promoter of Bax, and then lead to the cleavage of caspase 3, which mediated apoptosis.

Our study is the first to clarify the expression and role of endogenous microRNA-146a after MIRI. We also found one new target gene of microRNA-146a, Med1, which might mediate apoptosis through regulating the p53 related signal pathway in MIRI. However, more studies are still needed to verify the apoptosis signal pathway in this process.

## Conclusion

In conclusion, microRNA-146a may demonstrate compensatory upregulation in the early stage of MIR, and it possesses a protective effect against MIRI, which might be partially mediated by the target gene Med1 and related apoptosis signal pathway. Thus, it is suggested to increase the expression of microRNA-146a at the initial stage of myocardial ischaemia reperfusion as early as possible through interventions, such as drugs, surgery or direct exogenous addition of microRNA-146a mimics, to better protect the heart.

## Data Availability

The datasets used and/or analysed during the current study are available from the corresponding author on reasonable request.

## Abbreviations

AAR: Ratio of the area at risk
EF: Ejection fraction
FS: Fractional shortening
IS: Infarction size
KO: microRNA-146a deficient
LAD: Left anterior descending artery
LV: Left ventricle area
Med1: Mutated mediator complex subunit 1
MIR: Myocardial ischaemia reperfusion
MIRI: Myocardial ischaemia reperfusion injury
qRT-PCR: quantitative real-time PCR
WT: Wild type
